# GPCR-induced YAP activation sensitizes fibroblasts to profibrotic activity of TGFβ1

**DOI:** 10.1371/journal.pone.0228195

**Published:** 2020-02-13

**Authors:** Katarina Zmajkovicova, Yasmina Bauer, Katalin Menyhart, Marie Schnoebelen, Diego Freti, Maxime Boucher, Bérengère Renault, Rolf Studer, Magdalena Birker-Robaczewska, Axel Klenk, Oliver Nayler, John Gatfield

**Affiliations:** Idorsia Pharmaceuticals Ltd., Allschwil, Switzerland; University of Colorado School of Medicine, UNITED STATES

## Abstract

Tissue fibrosis is a pathological condition characterized by uncontrolled fibroblast activation that ultimately leads to organ failure. The TGFβ1 pathway, one of the major players in establishment of the disease phenotype, is dependent on the transcriptional co-activators YAP/TAZ. We were interested whether fibroblasts can be sensitized to TGFβ1 by activation of the GPCR/YAP/TAZ axis and whether this mechanism explains the profibrotic properties of diverse GPCR ligands. We found that LPA, S1P and thrombin cooperate in human dermal fibroblasts with TGFβ1 to induce extracellular matrix synthesis, myofibroblast marker expression and cytokine secretion. Whole genome expression profiling identified a YAP/TAZ signature behind the synergistic profibrotic effects of LPA and TGFβ1. LPA, S1P and thrombin stimulation led to activation of the Rho-YAP axis, an increase of nuclear YAP-Smad2 complexes and enhanced expression of profibrotic YAP/Smad2-target genes. More generally, dermal, cardiac and lung fibroblast responses to TGFβ1 could be enhanced by increasing YAP nuclear levels (with GPCR ligands LPA, S1P, thrombin or Rho activator) and inhibited by decreasing nuclear YAP (with Rho inhibitor, forskolin, latrunculin B or 2-deoxy-glucose). Thus, we present here a conceptually interesting finding that fibroblast responses to TGFβ1 can be predicted based on the nuclear levels of YAP and modulated by stimuli/treatments that change YAP nuclear levels. Our study contributes to better understanding of fibrosis as a complex interplay of signalling pathways and proposes YAP/TAZ as promising targets in the treatment of fibrosis.

## Introduction

Fibrotic diseases are major contributors to morbidity and mortality in the industrialized world, affecting almost every organ including lung, liver, kidney, heart or skin. Fibrosis is an uncontrolled wound healing process resulting from tissue injury, inflammation and fibroblast activation, finally leading to accumulation of extracellular matrix (ECM) components in tissues and to organ failure. The underlying mechanisms of fibrosis have been intensely studied and it has become clear that multiple pathways (inflammatory cytokine and chemokine signaling) regulate fibrotic diseases with a few central players, including WNT and TGFβ1 [1–3]. These pathways crosstalk to drive tissue fibrosis, exemplified by the necessity of multiple factors for induction of full fibrotic phenotype [4] and by several convergence points between TGFβ1, WNT and YAP signaling [3].

Recently, YAP and TAZ were described as essential components in the pathogenesis of fibrosis [3, 5–11]. YAP/TAZ are two highly related transcriptional coactivators, which are regulated by biomechanical forces, Hippo, Wnt and GPCR signalling as well as metabolic processes [12]. They exert their function via binding to TEADs [13] as well as other transcription factors [12]. Being potentially relevant to fibrosis, activated YAP/TAZ proteins are translocated to the nucleus where they crosstalk with the TGFβ pathway on a transcriptional level by binding to Smad2/3/4 complexes, as initially discovered in human and mouse embryonic stem cells, mammary epithelial cells, malignant mesothelioma and breast cancer cell lines [14–17]. It was shown that YAP/TAZ knockdown blocks the profibrotic effects of TGFβ1 [18, 19] and TGFβ2 [20] and that substrate stiffness, which impacts YAP/TAZ localization and activity also regulates the output of the TGFβ1 pathway [21].

We hypothesized that GPCR ligands, which regulate YAP/TAZ, might modulate the profibrotic responses to TGFβ1. We have recently shown that activation of G_αs_-coupled IP receptors on fibroblasts blocked the effects of TGFβ1 via inhibition of YAP/TAZ [18]. We thus became interested whether the opposite, i.e. activation of YAP by GPCR ligands, could sensitize fibroblasts to TGFβ1-induced fibrotic processes, as this had not been experimentally addressed before. Lysophosphatidic acid (LPA, binding to LPA_1_-_5_ receptors), sphingosine-1-phosphate (S1P, binding to S1P_1_-_5_ receptors) and thrombin (PAR1, 3 and 4 receptors) have been shown to activate YAP/TAZ in MCF10A and HEK293A cells [22–24]. At the same time, there is increasing evidence that these GPCR ligands have an important role in fibrosis. LPA is implicated in the pathogenesis of scleroderma, lung and kidney fibrosis [25–28] with LPA_1_ being the primary candidate mediating fibrotic effects [29, 30]. S1P was found to be elevated in idiopathic pulmonary fibrosis [31], human liver fibrosis [32] and is profibrotic in experimental models of lung fibrosis [33] mainly acting through S1P_2_ and S1P_3_ [34]. Interestingly, the profibrotic effects of thrombin [35–37] are independent of its role in hemostasis but dependent on the activation of protease-activated receptor 1 (PAR-1) [38]. However, for all of these ligands, the mechanistic connection between GPCR ligands, promotion of fibrosis and the potential role of YAP in this process is not completely understood.

The aim of our study was to investigate the link between GPCR signaling and the TGFβ1 pathway in fibrosis. We asked the question whether activation of the GPCR/YAP axis in fibroblasts would enhance the response to TGFβ1 in fibroblasts and whether this mechanism can contribute to the profibrotic properties of diverse GPCR ligands.

## Materials and methods

### Cell culture and treatments

Normal human foreskin fibroblasts (NHDF, Promocell C-123000, lot 0062308), normal human lung fibroblasts (NHLF #2512, Lonza, lot 543644) and normal human cardiac atrial fibroblasts (NHCF, Lonza #2903, lot 214476), were cultivated on gelatin coated plates in complete cell growth medium FBM supplemented with FGM-2 or FGM-3 SingleQuots (Lonza) up to passage 4. Starvation medium was prepared from FBM (Lonza) and contained 0.1% fatty acid free bovine serum albumin (BSA,Calbiochem) and penicillin/streptomycin. Cells were maintained at 5% CO_2_ and 37°C. Cells were treated with the following stimuli/compounds: TGFβ1 (Peprotech), Rho inhibitor I and Rho activator II (Cytoskeleton), lysophosphatidic acid (LPA), latrunculin B, thrombin, 2-deoxy-D-glucose (Sigma Aldrich) and sphingosine-1-phosphate (S1P, Enzo Life Sciences).

### Immunoblotting

Cells were washed with phosphate buffered saline (PBS) and then lysed on ice in RIPA buffer (Sigma Aldrich) containing Phosphatase inhibitor cocktail 2 (Sigma Aldrich), Complete protease inhibitor cocktail (Roche), 100mM NaF, 4mM Na_3_VO_4_, 1mM phenylmethylsulfonylfluoride (PMSF), 1mM dithiothreitol (DTT) and 100U/ml benzonase (Sigma Aldrich). Samples were resolved by SDS-PAGE on 4–12% Novex Bis-Tris precast gels (Thermo Fisher Scientific) and analyzed by western blotting using the following antibodies: fibronectin sc-6952 (Santa Cruz Biotechnology), YAP #14074, TAZ #4883, Smad2 #5339, tubulin #2148, phospho-YAP (Ser127) #13008 (Cell Signaling Technologies), αSMA ab5694, GAPDH ab9485, Smad3 ab40854, phopsho-Smad3 (S423+S425) ab52903 (Abcam), collagen type I OAMA03716, collagen type III OASB02204 (Aviva), vimentin #M7020 (Dako Cytomation) and HRP-coupled secondary antibodies (GE Life Sciences, Thermo Fisher Scientific). Membranes were treated with Western Lightning Enhanced Chemiluminescence Substrate (Perkin Elmer), and the chemiluminescence signal was recorded by the chemiluminescence reader Fusion FX6 (Vilber-Lourmat). Signals were quantified using densitometric analysis in ImageJ software.

### Subcellular fractionation and immunoprecipitation

Subcellular fractionation was performed as described [18]. In detail, primary dermal fibroblasts were seeded in 15cm cell culture dishes and grown to 80% confluency. For fractionation of nuclear and cytoplasmic proteins, cells were lysed in 1ml of buffer A (10 mM HEPES pH 7.9, 10 mM KCl, 0.1 mM EDTA, 1 mM DTT, Phosphatase inhibitor cocktail 2 (Sigma Aldrich), Complete protease inhibitor cocktail (Roche), 100mM NaF, 4mM Na_3_VO_4_, 1mM phenylmethylsulfonylfluoride (PMSF)) for 15 min on ice. Nonidet-P40 was added to a final concentration of 0.5%. Samples were centrifuged at 13’000 g for 1 min at 4°C. Nuclear pellets were washed with buffer A, resuspended in 250μl of buffer B (20 mM HEPES, pH 7.9, 400 mM NaCl, 1mM EDTA, 1mM DTT, Phosphatase inhibitor cocktail 2 (Sigma Aldrich), Complete protease inhibitor cocktail (Roche), 100mM NaF, 4mM Na_3_VO_4_, 1mM PMSF) and incubated for 15 min on ice. Lysates were cleared by centrifugation at 13’000 g for 10 min at 4°C and the supernatant was kept as nuclear fraction. For immunoprecipitation experiments, the nuclear fractions were subjected to immunoprecipitation with Smad2/3 (#610843, BD Transduction Laboratories) antibodies added at 1/100 dilution. Protein G-Sepharose beads (Sigma Aldrich) were added after 5h and incubated for additional 1h with the samples. Beads were washed thrice with buffer B, immunoprecipitated proteins were eluted by addition of 2-fold concentrated sample buffer and heated to 70°C for 10 min.

### YAP immunofluorescence imaging and high content analysis

YAP imaging studies were performed as described [18]. Cells were seeded in gelatin-coated CellCarrier Ultra 384-well plates (Perkin Elmer) at the density of 2’500 cells/well. After experimental treatment, the cells were fixed with 3% Paraformaldehyde/Phosphate buffered saline (PFA/PBS) for 15 min, washed once with PBS, permeabilized with 0.1% Triton-X100 in PBS for 10 min and blocked with 4% BSA/PBS for 1h. Primary antibodies against YAP (1/400, #14074, Cell Signaling technology) were diluted in 4% BSA/PBS and incubated with the cells overnight at 4°C. Cells were then washed thrice with PBS/0.25% Tween20 and incubated with secondary antibodies Alexa488 goat anti-rabbit (A11034, Invitrogen) diluted 1/500 in 4% BSA/PBS with Hoechst 33342 (1μg/ml, Invitrogen) for 1h. After washing cells 2x with PBS/0.25% Tween20 and 1x with PBS, cells were imaged on Opera Phenix High Content Screening System (Perkin Elmer) using 20x water immersion objective and confocal mode. Images were analyzed in Harmony High-Content Imaging and Analysis Software (Perkin Elmer). The nuclear intensity of YAP staining was calculated as a sum of IntensityAlexa488 in the nuclear region (defined by Hoechst staining). The values were normalized to control conditions. In each experiment, 1’000–4’000 cells were counted per experimental condition.

### Proximity ligation assay

Fibroblasts were seeded in gelatin-coated CellCarrier Ultra 384-well plates (Perkin Elmer) at the density of 2’500 cells/well. After experimental treatment, the cells were fixed with 4% PFA/PBS for 15 min, washed once with PBS, permeabilized with 0.1% Triton-X100 in PBS for 10 min. The Duolink Proximity ligation assay procedure was performed according to manufacturer’s instructions (Sigma-Aldrich). YAP antibody (sc-101199, Santa Cruz) and Smad2 antibody (#14074, Cell Signaling Technology) were used at 1/500 dilution overnight at 4°C. Nuclei were counterstained with DAPI. Cells were imaged on Opera Phenix High Content Screening System (Perkin Elmer) using 63x water immersion objective and confocal mode. Images were analyzed in Harmony High-Content Imaging and Analysis Software (Perkin Elmer). The nuclear region was defined based on the DAPI staining and the number of spots per nucleus was determined by the software. A minimum of 200 cells per condition and per experiment were evaluated.

### ELISA

Fibroblasts were seeded at a density of 10’000 cells/well onto gelatin-coated 96-well plates in 100 μl/well complete growth medium. After experimental treatment, the concentrations of PAI-1 in the cell supernatant were determined using the Human Serpin E1/PAI-1 Quantikine ELISA Kit (R&D Systems) according to manufacturer’s instructions. The absorbance was recorded on a Synergy4 microplate reader (BioTek Instruments).

#### [^3^H]-proline incorporation assays

Proline incorporation assays were performed as described [18]. Fibroblasts were seeded at a density of 10’000 cells/well onto gelatin-coated 96-well plates in 100 μl/well complete growth medium. The next day the medium was exchanged for 100 μl/well starvation medium. After 24h incubation, cells were treated and supplemented with 10 μl/well of L-[2,3-^3^H]-proline in starvation medium (0.2 μCi/well, Perkin Elmer) at the same time and cultivated for 24h. For determination of [^3^H]-proline incorporation, cell supernatants were discarded, cells were lysed in 150 μl/well NaOH (0.15 M) and lysates were incubated on ice for 30 min. For protein precipitation, 100 μl/well of trichloroacetic acid (50% (w/v) stock, final concentration 20%) was added and the lysates were incubated on ice for 30 min. Precipitated proteins were collected with the Filtermate cell harvester (Perkin Elmer) onto glass fiber filters (Unifilter-96, GF/C) (Perkin Elmer), filters were washed eight times with deionized water, dried and then supplemented with 60 μl/well of liquid scintillator Microscint20 (Perkin Elmer). Plates were subjected to liquid scintillation counting using TopCount (Perkin Elmer).

#### RNAi

Fibroblasts were transfected with following siRNAs: control siRNA targeting GFP (EHUEGFP, Sigma Aldrich), siRNA targeting WWTR1/TAZ (EHU08032, Sigma Aldrich), siRNA targeting YAP1 (EHU113021, Sigma Aldrich). Final siRNA amount used per well was 1pmol per well. Transfection complexes were prepared with Lipofectamine RNAiMax (Life Technologies) according to the manufacturer’s instructions. Transfection mixture was added to the bottom of the well and cells were plated on the top in 100 μl complete medium to perform reverse transfection. Fibroblasts were incubated 24h with the transfection complexes and then subjected to the experimental treatments.

### RT-qPCR

For RT-qPCR, 10’000 cells were seeded in gelatin-coated 96-well plate and grown for 24h. Medium was exchanged for starvation medium for an additional 24h. The cells were then subjected to experimental treatments for 3h. Cell lysis and reverse transcription was performed according to the TaqMan Fast Cells-to-Ct protocol (Thermo Fisher Scientific). The cDNA was preamplified according to the preamp kit with 14 cycles (Thermo Fisher Scientific). qPCR was performed on IFC gene expression chips on a Biomark HD (Fluidigm) using the following Taqman assays (Thermo Fisher Scientific, Applied Biosystems):

**Table:** 

Gene name	Probe number
EDN1	Hs00174961_m1
CTGF	Hs00170014_m1
CYR61	Hs00155479_m1
SERPINE1	Hs01126604_m1
IL11	Hs01055413_g1
GBP1	Hs04261213_m1
DDAH1	Hs00201707_m1
PMEPA1	Hs00375306_m1
CLDN4	Hs00533616_s1
EGR2	Hs00166165_m1
GNA12	Hs02863396_m1
GNA13	Hs01116111_m1
B2M	Hs00984230_m1
GUSB	Hs00939627_m1
HPRT1	Hs02800695_m1
PGK1	Hs00943178_g1
PPIA	Hs04194521_s1
GAPDH	Hs02758991_g1
YWHAZ	Hs03044281_g1

Results were calculated with the delta delta Ct method using B2M, GUSB, HPRT1, PGK1, PPIA, GAPDH and YWHAZ as housekeeping genes selected with normalization software GENORM [39]. The results were then transformed into expression values on a linear scale where a value of 1 reflects the limit of detection (= 30 cycles on Biomark HD). This method allows a comparison of expression of different genes on a linear scale [40].

### Gene expression microarray

**Fig 1:**
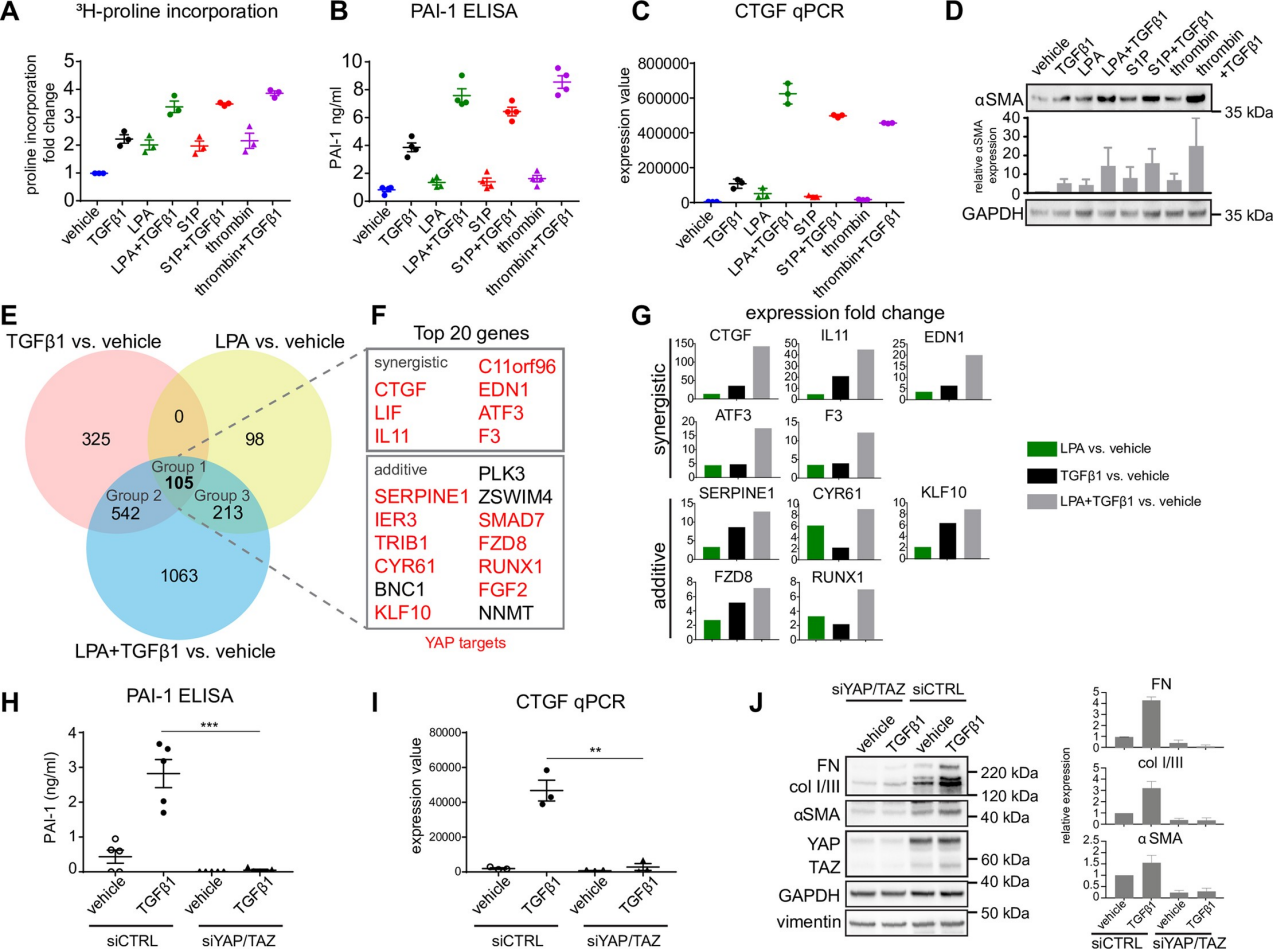
GPCR ligands and TGFβ1 cooperate to induce a profibrotic phenotype and YAP/TAZ target gene expression in NHDF. (A, B) NHDF were starved and stimulated with 5ng/ml TGFβ1, 1μM LPA, 1μM S1P, 1mU/ml thrombin alone or in combination for 24h. ECM synthesis was measured by ^3^H-proline incorporation (n = 3) and PAI-1 in the cell supernatant was determined by ELISA (n = 4). Mean+/- SEM. (C) NHDF were treated as in A for 3h. CTGF expression was determined by RT-qPCR (n = 3). Mean+/- SEM. Statistics for A-C are summarized in S6 Table. (D) NHDF were treated as in A. Levels of αSMA were determined by western blot. GAPDH served as loading control. The signal for αSMA was measured by image densitometry and expressed as relative value compared to vehicle-treated sample (n = 4). Mean+/- SEM. (E) Whole genome expression analysis of NHDF treated for 3h as indicated. Graphical representation of the Venn diagram analysis results. (F) Top 20 upregulated genes (when ordered by log2FC in the TGFβ1/LPA vs vehicle contrast) from Group 1 are depicted and classified for synergistic or additive effects of the TGFβ1/LPA combination. Genes highlighted in red represent YAP/TAZ target genes. (G) Graphs showing gene expression fold change values for selected genes from G for the indicated contrasts. (H) NHDF were transfected with control siRNA (siCTRL) or siRNAs against YAP/TAZ, starved and stimulated with 5ng/ml TGFβ1 for 24h. PAI-1 in cell supernatant was determined by ELISA (n = 5). Mean+/- SEM. (I) NHDF were treated as in H for 3h. CTGF gene expression was measured by RT-qPCR (n = 3). Mean+/- SEM. (J) NHDF were treated as in C. Levels of FN, collagen I/III, αSMA, YAP and TAZ were determined by western blot. GAPDH served as loading control. Images are representative of 3 independent experiments. Right panel shows results of image densitometry. FN, collagen and αSMA were normalized to the loading control. Relative expression to siCTRL vehicle samples are shown. n = 4, mean +/- SEM.

NHDF were seeded in 24-well plates (100’000 cells per well), grown overnight and starved for 4h before the experimental treatment. Cells were treated with vehicle control, 1μM LPA, 5ng/ml TGFβ1 or both for 3h and then lysed in TriReagent (Ambion). The experiment performed with 4 biological replicates for each treatment. Total RNA was isolated from the cell lysate using MagMax 96 microarray kit (Life Technologies) according to the manufacturer's instructions. Amplification and labeling were performed with Agilent Low RNA Input Quick Amp Labeling Kit according to the manufacturer’s instructions using 50 ng of total RNA as starting material. in A two color protocol was used, Human RNA reference (Agilent Stratagen) was labeled in Cy5 and all the samples were labeled in Cy3. The 8×60 K multiplex arrays with 50,599 60-mer oligonucleotides directed against the human transcriptome were used (SurePrint G3 Human GE 8x60k v3, Ref. G4851B, design ID 039494, Agilent). Each Cy3 samples (300 ng) were hybridized against the cRNA Human Ref_Pool_Cy5 (300 ng) that was used as internal calibrator for normalization. Hybridizations and washes were performed following the manufacturer’s instructions. The arrays were scanned using the Agilent microarray scanner (Ref. G2565BA) and Scan Control software 8.5.1, data were extracted with the Feature Extraction software 11.5.1.1. Microarray raw data were normalized using vsn (variance-stabilizing normalization), a method that enables within-array and between-array normalizations [41]. The dataset is available online from the Gene Expression Omnibus (http://www.ncbi.nlm.nih.gov/geo) with accession number GSE125519. The empirical Bayes method from the limma package [42] was used to compute moderated t-statistics and the corresponding p-values to identify differentially expressed genes in the following contrasts: (1) TGFβ1+vehicle versus vehicle, (2) LPA versus vehicle and (3) TGFβ1+LPA versus vehicle. The p values were corrected with the Benjamini and Hochberg's false discovery rate (FDR) controlling procedure [43] (F). We generated Venn diagrams to classify the responses to the different stimuli and generate several groups (group 1 to 3 as defined in Fig 1E) based on the responses. We further analyzed group 1 and partitioned it as follows: if the expression change in the combination treatment was at least 50% greater than the sum of the expression changes in the individual treatments, it was considered synergistic; if it was at least 50% less, it was considered antagonistic. Otherwise we assumed that the combination treatment was additive.

GSEA v3.0 (http://software.broadinstitute.org/gsea/index.jsp) software [44, 45] was used to investigate the enrichment of the YAP/TAZ target gene signature [46] in the following phenotypes/contrasts: TGFβ1+LPA vs vehicle, TGFβ1 vs vehicle and LPA vs vehicle. The enrichment was considered significant when the p value was <0.05.

### Statistical analysis and data reproducibility

Statistical analysis was performed using GraphPad Prism software. p values < 0.05 were considered statistically significant and set as follows: *—p< 0.05; **—p < 0.01; ***—p < 0.001. Unpaired 2-tailed t test was used in most cases, one sample 2-tailed t test was used when data was normalized to control sample (H_0_: sample means are equal to 1). The number of independent experiments (n) is stated in each figure legend.

## Results

### GPCR ligands LPA, S1P and thrombin cooperate with TGFβ1 to induce fibrotic responses in human dermal fibroblasts

To examine whether TGFβ1 signalling and GPCR activation cooperate in fibrotic processes, we assessed the effects of TGFβ1 and selected GPCR ligands, LPA, S1P or thrombin, on well-established fibrotic readouts in normal human primary dermal fibroblasts (NHDF). ECM synthesis, measured by ^3^H-proline incorporation, expression of secreted profibrotic factors, PAI-1 and CTGF, and αSMA upregulation, as a marker of fibroblast-to-myofibroblast transition were determined. TGFβ1 stimulation (5 ng/ml) increased ECM synthesis, PAI-1, CTGF and αSMA expression (Fig 1A–1D, S1A-C, statistics for 1A-C and S1A-C are summarized in S6 Table). The individual GPCR ligands, used at concentrations that are known to activate their target receptors, also elevated ECM synthesis (Fig 1A), but on their own had only modest effects on PAI-1 secretion and CTGF transcription (Fig 1B and 1C). The combination of TGFβ1 and either LPA, S1P or thrombin additively induced higher ^3^H-proline incorporation (Fig 1A) and αSMA levels compared to respective single treatment (Fig 1D). Most interestingly, whilst having only minor effects on PAI-1 secretion and CTGF expression on their own, LPA, S1P and thrombin synergistically augmented the effects of TGFβ1 (Fig 1B and 1C). Similar results were obtained when using 2 ng/mL of TGFβ1. Furthermore, in the presence of an Alk5 blocker TGFβ1 alone or in combination did not increase ^3^H-proline incorporation, PAI-1 secretion and CFTF mRNA levels, while the activity of GPCR agonists remained unaltered. These findings exclude an activation of TGF-β receptors by GPCR agonists at any point in the analyzed fibrotic processes (S1A–S1C Fig).

We concluded that LPA, S1P and thrombin signaling cooperates with the TGFβ1 pathway to establish a fibrotic response in dermal fibroblasts. These two pathway types act in additive/synergistic manner on several aspects of the profibrotic phenotype (ECM and cytokine synthesis) and thus may regulate the same target genes by cooperating mechanisms.

### Whole genome expression analysis reveals LPA-TGFβ1 synergy in inducing the expression of multiple pro-fibrotic genes and a YAP/TAZ signature

Next, we analysed the effect of the two stimuli, TGFβ1, LPA and their combination, on the transcriptional level in NHDF and looked at the YAP/TAZ signature in the overall gene expression change. We used a GSEA approach to look for enrichment of YAP/TAZ target genes [46] and could observe an upregulation of this gene set in the combination treatment as well as in the individual treatments (S1D Fig) which confirms the relevance of YAP/TAZ signalling in the fibrotic response.

Linear models, which were fitted to the log2-transformed expression levels, were applied to identify differentially expressed genes that were defined by a differential expression greater than 0.7 and a FDR-adjusted P value less than 0.005 in the single (TGFβ1 or LPA) or combination (TGFβ1 + LPA) treatment versus vehicle. The Venn diagram (Fig 1E) depicts a total of 2346 genes that were differentially expressed in at least one of the conditions compared to vehicle including, 972 genes modulated by TGFβ1 (for more detailed analysis of these genes see S2 Fig; S1 Table). The Venn diagram depicts 105 genes (Group 1) that were differentially expressed in both single treatment arms (TGFβ1 or LPA) and in combination treatment (TGFβ1 + LPA; S2 Table). Group 2 represents 542 genes that were differentially expressed in both TGFβ1 and TGFβ1+LPA treatment but they remained unaffected by LPA treatment (S3 Table). Finally, Group 3 corresponds to 213 genes that were differentially expressed in both LPA treatment and TGFβ1 + LPA treatment, but showed no change in the TGFβ1 treatment (S4 Table). We next selected a few prototypic genes from the individual groups; CTGF, IL11, EDN1, SERPINE1 and CYR61 from Group 1, PMEPA1, CLDN4 and EGR2 from Group 2, and GBP1 and DDAH1 from Group 3. Their gene microarry expression profiles were confirmed in RT-qPCR experiment (S1E Fig).

We then focused our analysis on the top 20 upregulated genes of Group 1 to further analyze differences of gene expression of the combination treatment (TGFβ1 + LPA) versus single treatment (LPA or TGFβ1) and defined 3 classes of effects, i.e. synergistic, antagonistic and additive, using a 50% change threshold as defined in materials and methods. All 20 genes had synergistic (7) or additive (13) behaviour.

Interestingly, all 7 genes that were classified as synergistic were YAP/TAZ targets (Fig 1F, S2 Table) and 9/13 of the genes classified as additive (Fig 1F, S2 Table) were also shown to be regulated by YAP/TAZ [13, 46–50]. Among these genes, CTGF [51, 52], IL11 [53], EDN1 [54], SERPINE1/PAI-1 [55], F3/tissue factor [56], CYR61 [57], KLF10 [58], ATF3 [59], FZD8/Frizzled-8 [59] and RUNX1 [60] have been shown to be important profibrotic mediators. Gene expression profiles of these genes are shown in Fig 1G.

These results suggested that LPA and TGFβ1 signalling converges on YAP/TAZ and that YAP/TAZ could represent a central node to regulate fibrotic responses. To confirm this, siRNA experiments were conducted. Due to their redundant function [49], both YAP and TAZ were knocked down simultaneously. Knockdown of YAP/TAZ did not only completely block PAI-1 and CTGF (YAP/TAZ targets), αSMA, collagen I/III and fibronectin (bona fide fibrotic readouts) upregulation in response to TGFβ1, but even decreased their basal expression in the absence of TGFβ1 (Fig 1I and 1J).

Summarizing, we show that YAP target gene expression in fibroblasts is a central feature of the TGFβ1/LPA response and that YAP/TAZ are indispensable for TGFβ1-induced fibrotic processes.

### LPA, S1P and thrombin activate YAP, increase YAP-Smad2 interaction in the nucleus and augment fibroblast responses to TGFβ1

Based on published data from HEK293T and MCF10 cells [23], we hypothesized that the expression of YAP target genes and their enhancement by the TGFβ1/LPA combination might be a result of YAP/TAZ activation by LPA, or more generally a pro-fibrotic GPCR agonist.

**Fig 2:**
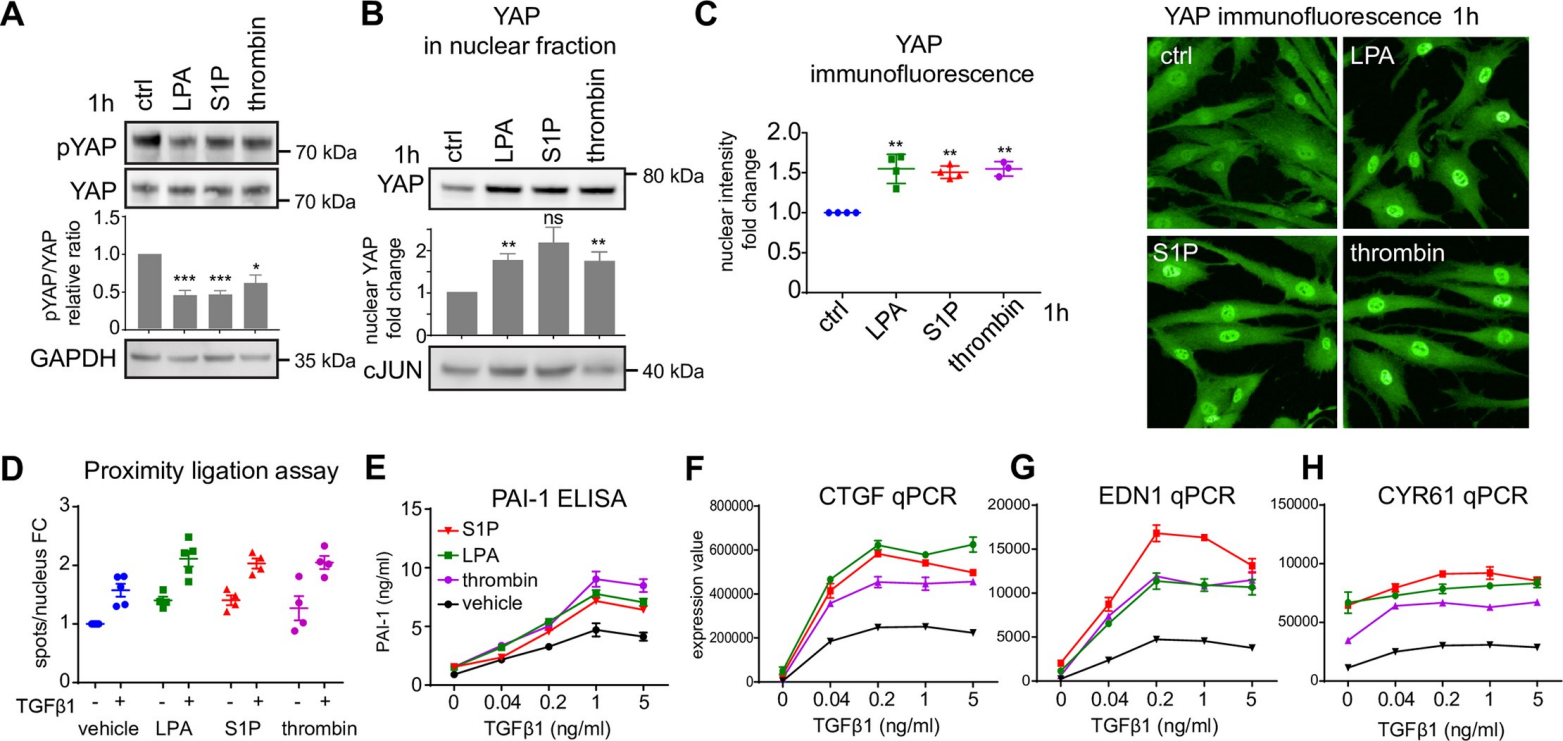
GPCR ligands activate YAP to enhance TGFβ1 response. (A) NHDF were starved and stimulated with 1μM LPA, 1μM S1P or 1mU/ml thrombin for 1h and the whole cell lysates were analyzed by western blot for pS127 YAP, YAP and GAPDH. Representative images are shown. The western blots were quantified by image densitometry. The pYAP signal was normalized to total YAP and expressed as fold change relative to vehicle-treated samples (n = 4–6, biological replicates). Mean+/- SEM. One-sample t-test against the normalized value 1 (vehicle) was performed. (B) NHDF were treated as in A and the level of YAP was determined in nuclear fractions. Representative images are shown. The western blots were quantified by image densitometry. The YAP signal was expressed as fold change relative to vehicle-treated samples (n = 3–4, biological replicates). Mean+/- SEM. cJUN was used as a control for nuclear fraction. One-sample t-test against the normalized value 1 (vehicle) was performed. (C) NHDF were treated as in A. The nuclear intensity of YAP was analyzed by high content imaging of cells stained with anti-YAP antibody. Results were normalized to vehicle-treated cells. Example images are shown on the right (n = 3–4). Mean+/- SEM. One sample t-test was performed comparing normalized treatment effects to value 1 (vehicle). (D) NHDF were starved and stimulated for 1h with 1μM LPA, 1μM S1P or 1mU/ml thrombin with or without 5ng/ml TGFβ1. The interaction between nuclear YAP and Smad2 was monitored by proximity ligation assay. Results were normalized to vehicle-treated cells. FC—fold change (n = 4). Mean+/- SEM. Statistics are summarized in S6 Table. (E) NHDF were starved and stimulated for 24h with an increasing concentration of TGFβ1 alone or in combination with 1μM LPA, 1μM S1P or 1mU/ml thrombin. PAI-1 in cell supernatant was determined by ELISA (n = 3). Mean+/- SEM. (F-H) NHDF were starved and stimulated for 3h as in G. CTGF, EDN1 and CYR61 expression was determined by RT-qPCR (n = 3). Mean+/- SEM.

We therefore analysed the effects of LPA as well as S1P and thrombin on YAP at the cellular level by immunoblotting and immunofluorescence microscopy. We focused our analysis on YAP protein due to the availability of good phosphorylation-specific antibodies as well as due to the overlapping functions of YAP/TAZ and their parallel regulation by upstream pathways [49, 61]. In NHDF, all three GPCR agonists decreased the S127 YAP phosphorylation by ~50% already 1h after stimulation (Fig 2A), indicating YAP activation, and at the same time the nuclear levels of YAP increased by 50–100%, measured by subcellular fractionation and high content imaging (Fig 2B and 2C). Nuclear translocation induced by LPA was rapid, reached peak levels at 1h and persisted at least 6 hours (S3A Fig). As expected [18], treatment with TGFβ alone did not affect nuclear levels of YAP (S3B Fig) and the phosphorylation of YAP, while it did lead to Smad3 phosphorylation (S3C Fig). This shows that although TGFβ1 requires YAP for pro-fibrotic responses TGFβ1 does not actually change the YAP activation status. The increase of nuclear YAP functionally translated to upregulation of YAP/TAZ target gene expression 3h after stimulation, as revealed in the enrichment plot of LPA-stimulated NHDFs (S1D Fig). YAP is known to interact with Smad2/3 and to enhance the transcription of combined YAP/Smad target genes [14–17]. Therefore, we wanted to analyse whether GPCR-stimulated YAP activation in conjunction with TGFβ1 leads to an increase in YAP/Smad2 interactions in fibroblasts. We used a proximity ligation assay (PLA) to quantify the number of YAP and Smad2 interactions in the nucleus. This assay revealed that the number of YAP-Smad2 interactions increased in TGFβ1-only condition (due to increase in nuclear Smad2) or upon GPCR ligand (LPA, S1P or thrombin) addition (due to increase in nuclear YAP) compared to the baseline. The number of complexes further increased with combined treatment of TGFβ1 and GPCR ligands (Fig 2D, statistics are summarized in S6 Table). In line with the PLA data, immunoprecipitation confirmed that the levels of nuclear YAP/Smad2/3 complexes were increased upon LPA addition in TGFβ1-treated NHDFs (S3D Fig).

Based on these results we hypothesized that for fibrotic readouts, which are regulated by both the TGFβ1/Smad pathway and YAP in a synergistic or additive manner (Group 1 genes), GPCR-induced YAP activation could augment the cellular response to TGFβ1. To show this experimentally, we treated NHDFs with increasing concentrations of TGFβ1 in the absence or presence of a constant concentration of LPA, S1P or thrombin, followed by measurements of secreted PAI-1 (ELISA) and quantification of CTGF, EDN1 and CYR61 mRNA levels. While the GPCR agonists by themselves had little effect on PAI-1 secretion or CTGF/EDN1 mRNA expression, their presence as co-stimulants augmented the response to TGFβ1 in these three readouts resulting in a 2- to 5-fold amplified response across all TGFβ1 concentrations and E_max_ values reaching 180% to 550% compared to the E_max_ values in the absence of co-stimulants while EC_50_ values for TGFβ1 with the different co-treatments remained rather constant (Fig 2E and 2F and S5 Table). For the CYR61 expression, the GPCR ligands themselves already increased mRNA levels and additive effects between GPCR ligands and TGFβ1 were observed (Fig 2H). In contrast, no changes in the response to TGFβ1 were observed for the Group 2 genes and known Smad2/3-only targets [62] PMEPA1, EGR2 and CLDN4 (S3E Fig). Taken together, stimulation of fibroblasts with LPA, S1P or thrombin leads to rapid YAP activation, an increase in nuclear YAP/Smad2 interaction, and a synergistic or additive augmentation of fibroblast responses to TGFβ1 which by itself does not affect YAP activation.

### LPA, S1P and thrombin regulate YAP-induced profibrotic responses via Rho activation

**Fig 3:**
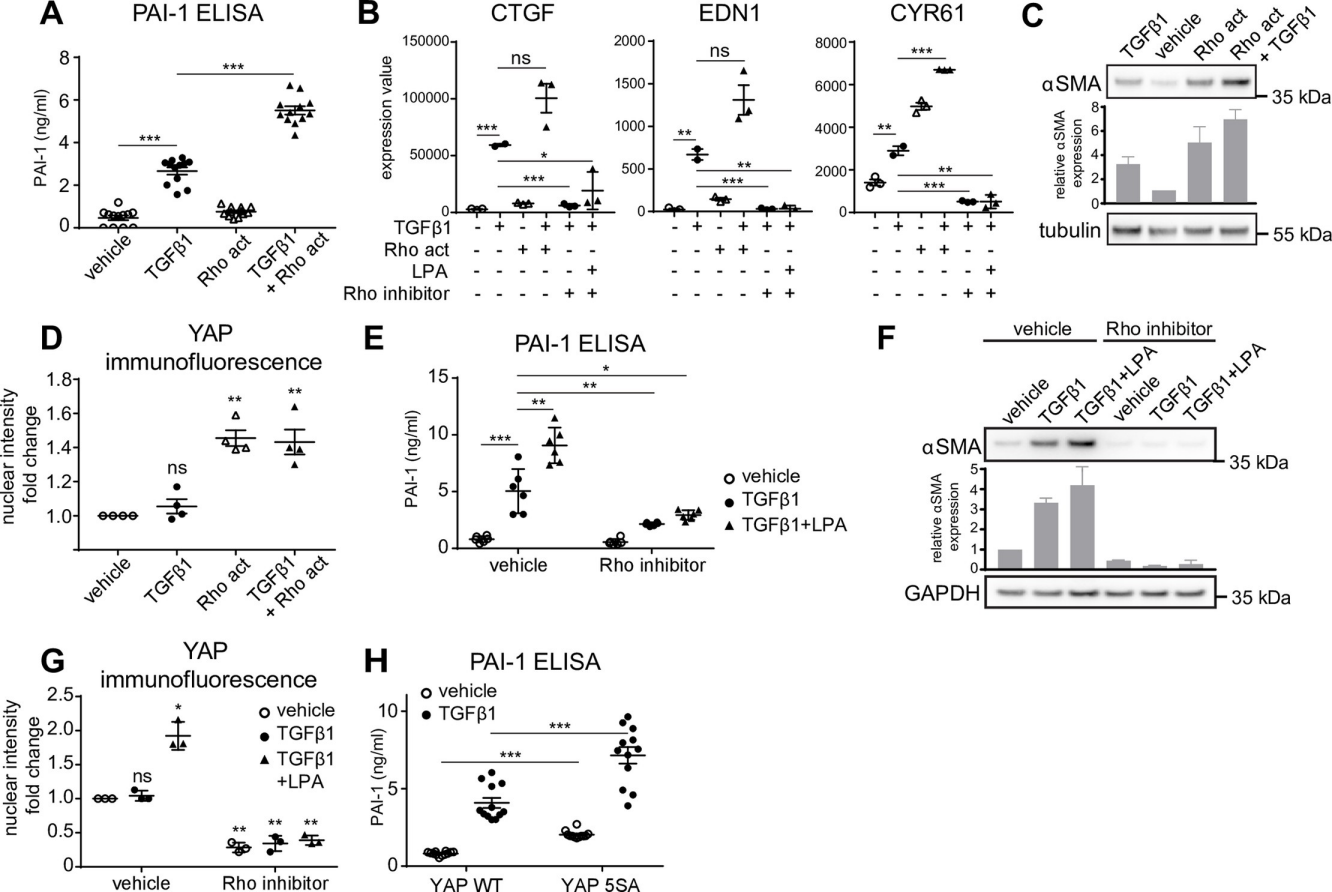
GPCR ligands regulate TGFβ1 response via the Rho/YAP axis. (A) Human dermal fibroblasts were starved and stimulated with 5ng/ml TGFβ1, 1ng/ml of Rho activator (Rho Act) or a combination of the two. PAI-1 levels in the cell supernatant were measured by ELISA after 24h (n = 9). Mean+/- SEM. (B) CTGF, EDN1 and CYR61 expression was measured by RT-qPCR after 3h in NHDF treated as indicated (n = 3). Mean+/- SEM. (C) αSMA levels were determined by western blot at 24h in cells treated as in A. The signal for αSMA was measured by image densitometry and expressed as relative value compared to vehicle-treated sample (n = 2). Mean+/- SEM. (D) NHDF were starved and stimulated with 5ng/ml TGFβ1, 1ng/ml of Rho activator (Rho Act) or a combination of the two for 1h. The nuclear intensity of YAP was analyzed by high content imaging of cells stained with anti-YAP antibody. Results were normalized to vehicle-treated cells (n = 4). Mean+/- SEM. One sample t-test was performed comparing normalized treatment effects to value 1 (vehicle). (E, F) NHDFs were starved, pretreated with Rho inhibitor (2ng/ml) and stimulated with 5ng/ml TGFβ1, 1μM LPA or the combination. PAI-1 levels in the cell supernatant were measured by ELISA after 24h (n = 6). Mean+/- SEM. αSMA levels were determined by western blot at 24h. The signal for αSMA was measured by image densitometry and expressed as relative value compared to vehicle-treated sample (n = 2). Mean+/- SEM. (G) NHDFs were starved, pretreated with Rho inhibitor (2ng/ml) and stimulated with 5ng/ml TGFβ1, 1μM LPA or the combination for 1h. The nuclear intensity of YAP was analyzed by high content imaging of cells stained with anti-YAP antibody. Results were normalized to vehicle-treated cells (n = 3). Mean+/- SEM. One sample t-test was performed comparing normalized treatment effects to value 1 (vehicle). (H) NHDF were transfected with myc-YAP WT or myc-YAP5SA and stimulated with 5ng/ml TGFβ1 or vehicle. PAI-1 levels in the cell supernatant were measured by ELISA after 24h (n = 12). Mean+/- SEM.

Studies in HEK293A cells have shown that YAP/TAZ is activated by LPA, S1P or thrombin via Rho signalling [23, 24]. We wanted to demonstrate this in our fibrosis model and show that interventions at this level impact on the observed synergistic/additive effects on TGFβ1-induced profibrotic processes. To this aim, we used a Rho activator (CNF1 toxin-based [63]) and a Rho inhibitor (C3 toxin-based [64]). We show that the Rho activator–while being almost without effect on PAI1 levels on its own and thus reminiscent of the studied GPCR agonists—significantly enhanced TGFβ1-induced PAI-1 secretion (Fig 3A). Similarly, CTGF, EDN1 and CYR61 expression (Fig 3B) and αSMA levels (Fig 3C) were increased by the Rho activator in the combination treatment as compared to the TGFβ1 treatment alone, although the effects did not always reach statistical significance. This response augmentation correlated with an increased nuclear level of YAP (Fig 3D), which was again absent in the TGFβ1-only condition. Conversely, the Rho inhibitor blocked TGFβ1+LPA-induced PAI-1 induction (Fig 3E), CTGF, EDN1 and CYR61 expression (Fig 3B) and αSMA expression (Fig 3F). Notably, the Rho inhibitor also blocked the profibrotic effects of TGFβ1 itself (Fig 3B, 3E and 3F), and decreased even the basal levels of nuclear YAP (Fig 3G).

To directly confirm the role of nuclear YAP in augmenting TGFβ1 responses in NHDF, we transfected fibroblasts with a YAP mutant (YAP 5SA) that is predominantly localized to the nucleus. YAP 5SA has LATS phosphorylation site mutations and cannot be inactivated by LATS to be excluded from the nucleus [18, 65]. NHDFs that were transfected with YAP 5SA produced more PAI-1 compared to YAP WT-transfected cells under basal conditions and responded to TGFβ1 with higher PAI-1 secretion (Fig 3H).

In summary, we have shown that the ability of certain GPCR ligands to enhance the fibroblast response to TGFβ1 was mediated via the Rho/YAP axis.

### Levels of nuclear YAP predict profibrotic responses to TGFβ1

As shown above, YAP knockdown in NHDF essentially abolished the TGFβ1 responsiveness. Also, reduction of nuclear YAP in fibroblasts by other means i.e through stimulation of the cAMP pathway [18], attenuates the fibrotic responses to TGFβ1. In the present study we show that increasing nuclear YAP levels in fibroblasts augments TGFβ1-induced fibrotic responses.

To investigate if the magnitude of the profibrotic response quantitatively correlates with the levels of nuclear YAP, we treated NHDFs with a constant concentration of TGFβ1 in the presence of various co-stimuli that were expected to either positively or negatively modulate nuclear YAP levels. Increasing concentrations of the co-stimuli LPA, S1P and thrombin were used in NHDFs to gradually increase YAP nuclear levels. To decrease nuclear YAP and thus inactivate it, we used the actin depolymerizing agent latrunculin B [66], a Rho inhibitor [23], forskolin [18, 23] or 2-deoxy-D-glucose (2-DG) [67, 68].

**Fig 4:**
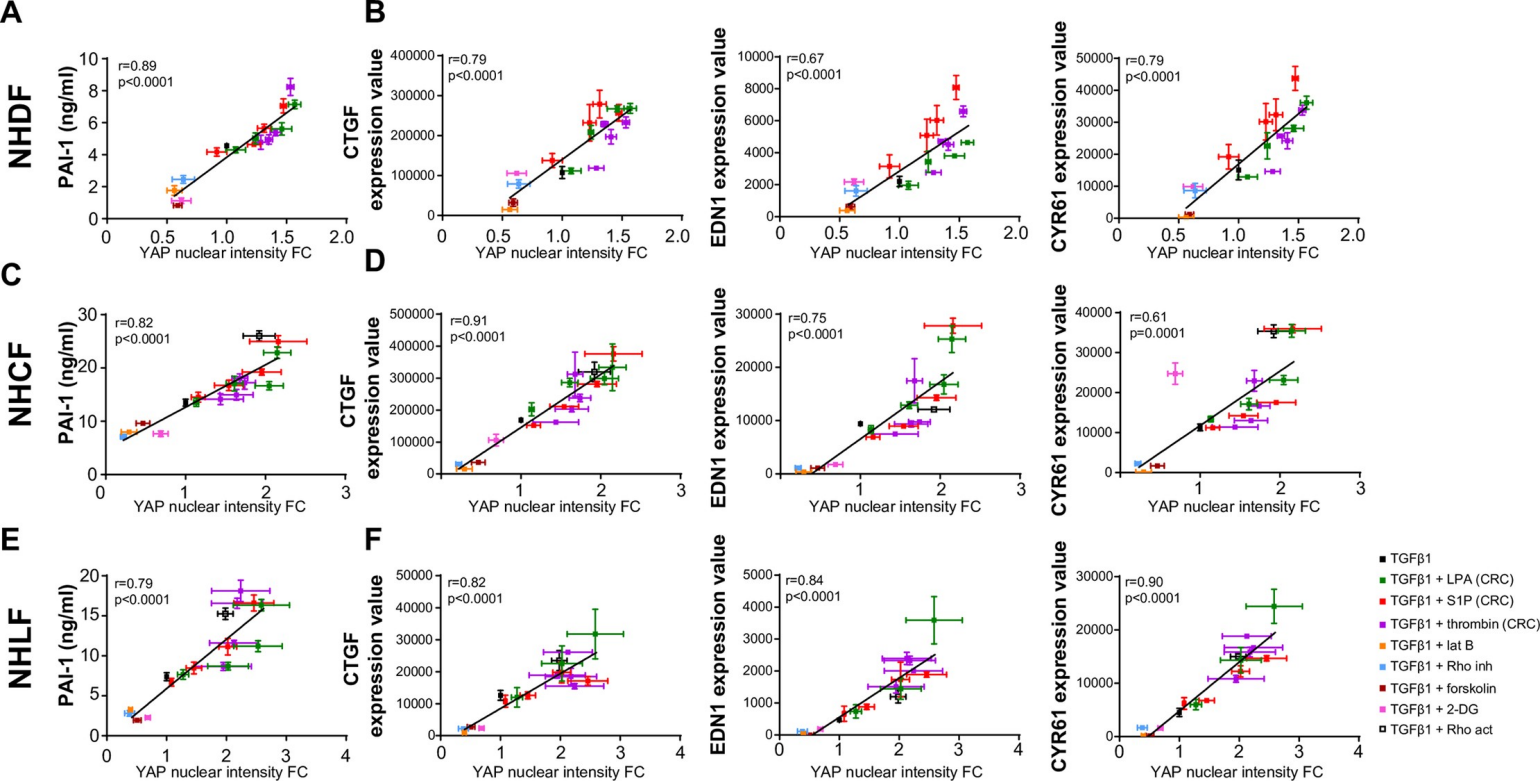
Response to TGFβ1 correlates with nuclear levels of YAP. (A-F) NHDF, NHCF and NHLF were starved and then treated with 5ng/ml TGFβ1 alone or in combination with indicated YAP modulators; concentration-response curve (CRC) of LPA and S1P (10-fold dilutions starting at 1μM), thrombin (10-fold dilutions starting at 1mU/ml), 500nM latrunculin B (lat B), 2ng/ml Rho inhibitor (inh), 10μM forskolin, 50mM 2-DG or 1ng/ml Rho activator (act). Integrated nuclear intensity of YAP was analysed after 1h by high content imaging analysis of cells stained against YAP and expressed as fold change (FC) versus TGFβ1-only treated cells. The same treatments were done for 3h to measure gene expression by RT-qPCR and for 24h to analyse PAI-1 in cell supernatants by ELISA. PAI-1 levels and gene expression were correlated (linear correlation) with YAP nuclear staining. (n = 3–4; YAP imaging). (n = 3; RT-qPCR). (n = 4–8; ELISA). Mean+/- SEM.

For the tested co-stimulus-TGFβ1 treatments, YAP nuclear levels correlated significantly with PAI-1 levels in the cell supernatant (Fig 4A) and with mRNA expression levels of CTGF, EDN1 or CYR61 (Fig 4B), and this was the case for negative as well as positive regulators of nuclear YAP levels. Furthermore, treatments that decreased nuclear YAP also inhibited stimulation of αSMA expression (S4A Fig). In contrast, expression changes of the TGFβ1/Smad-only regulated genes PMEPA1, CLDN4 and EGR2 did not occur with the co-stimulant treatment and thus their expression did not correlate with nuclear YAP levels in these experiments (S4B Fig). The positive correlation of nuclear YAP levels and magnitude of TGFβ1-induced fibrotic responses (PAI-I secretion, CTGF, EDN1 and CYR61 expression) was confirmed in fibroblasts from different tissues of origin, including normal human primary cardiac fibroblasts (NHCFs) and normal human primary lung fibroblasts (NHLFs) (Fig 4C–4F).

Thus, we found that negative as well as positive modulation of baseline nuclear YAP levels by a variety of unrelated stimuli quantitatively translated to a decreased or increased transcription of profibrotic YAP/Smad target genes, or in other words that the response to TGFβ1 could be predicted by the measured nuclear levels of YAP.

## Discussion

**Fig 5:**
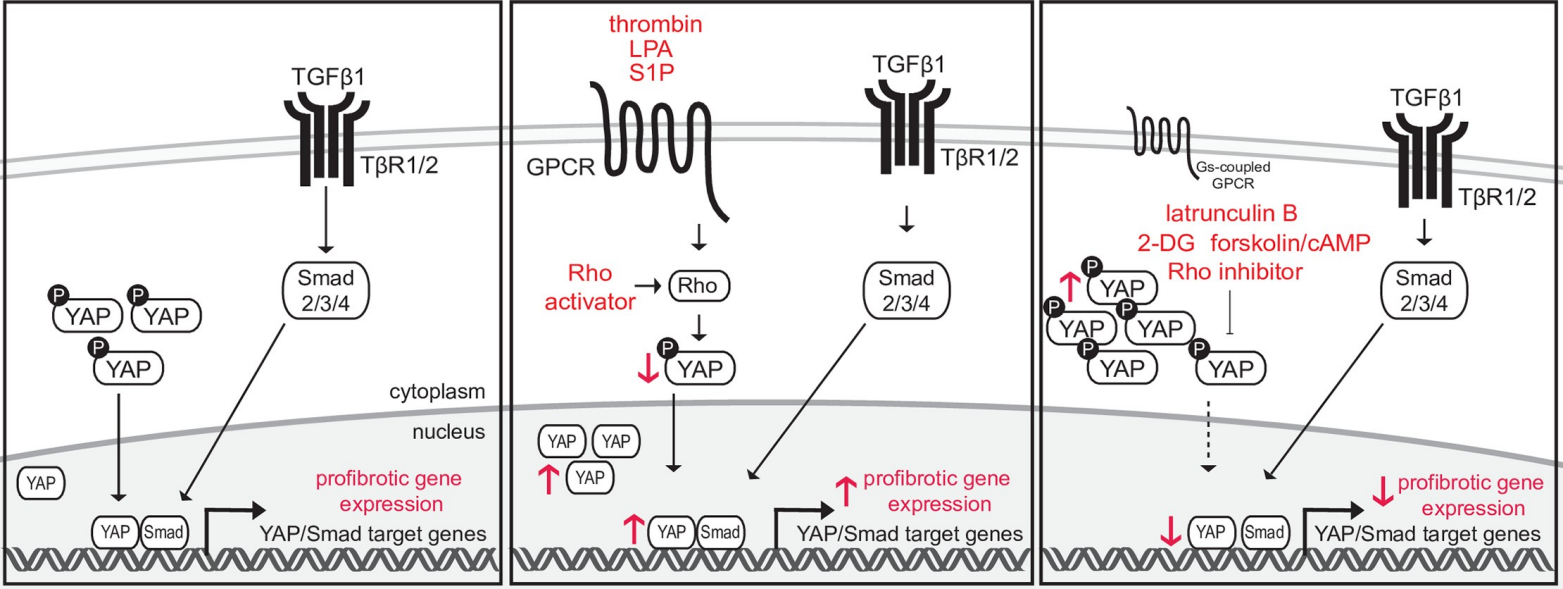
Schematic representation of the proposed mechanism. TGFβ1 leads to activation of Smad2/3/4 complexes and their nuclear translocation. In the nucleus, they are using YAP as a co-activator to induce transcription of YAP/Smad target genes, many of which are implicated in fibrogenesis. If GPCR ligands LPA, S1P or thrombin are present at the same time, they activate their receptors and trigger Rho activation. YAP is then dephosphorylated and accumulates in the nucleus. This increases the response to TGFβ1 by enhancing the expression of YAP/Smad-target genes. Conversely, latrunculin B, forskolin, Rho inhibitor, 2-DG or GPCR ligands increasing cAMP levels decrease nuclear levels of YAP and hereby decrease the expression of profibrotic YAP/Smad target genes in response to TGFβ1.

Our study provides novel mechanistic insights into the crosstalk between GPCR and TGFβ1 signaling in the context of tissue fibrosis. We show for the first time that LPA, S1P and thrombin activate YAP in fibroblasts and that this sensitizes the cells towards the profibrotic action of TGFβ1. The crosstalk between GPCR/YAP and TGFβ1/Smad pathways results in an increase of YAP/Smad complexes and in this way stimulates the expression of combined YAP/Smad target genes, many of which are important profibrotic mediators (Fig 5). We present here a conceptually interesting finding that GPCR ligands and other stimuli/treatments that have little pro-/anti-fibrotic effects by themselves can lead to sensitization/desensitization of fibroblasts to pro-fibrotic activities of TGFβ1, and that the intensity of these responses to TGFβ1 can be predicted based on the co-stimulants´ capability to positively or negatively modulate YAP nuclear levels. This concept of synergy is based on the fact that TGFβ1 requires YAP/TAZ to exert pro-fibrotic functions, but does not itself regulate YAP/TAZ presence in the nucleus.

We have previously shown that TGFβ1-induced fibrotic processes can be halted by inhibiting YAP via the IP receptor/G_s_/cAMP axis [18]. The current study builds on and extends these findings by activating GPCR receptors that stimulate YAP/TAZ and, on the opposite, aggravate the TGFβ1-induced phenotype. Our results provide mechanistic explanation why such divergent GPCR ligands as LPA, S1P [25], thrombin [38], serotonin [69], endothelin [54], histamine [70] and angiotensin II [71], play role in fibrotic processes in various tissues. Notably, many of them are known to activate YAP, i.e. LPA, S1P [23], thrombin [24], serotonin [72], endothelin [73] and angiotensin II [74]. This is in line with our hypothesis that a common pathway downstream of these receptors, namely the Rho/YAP/TAZ pathway, operates in the pathogenesis of fibrosis and that the fibrotic response of a particular tissue or cell type depends on its GPCR expression profile. This study does not exclude that other pathways downstream of GPCRs can contribute to fibrotic responses, such as the Rho/SRF/MRTF axis that actually has been also described to interact with YAP downstream of LPA and S1P [75, 76].

Due to their central role in fibrosis, YAP/TAZ might represent promising drug targets. In fact, the prominent pathologic effects of YAP/TAZ activation are already appreciated in cancer [77], and attempts to discover inhibitors of YAP/TEAD interaction for oncological indications are ongoing [78]. One of the other feasible strategies to target YAP in fibrosis would be to use antagonists of YAP-activating GPCRs. LPA antagonists are already in clinical development [79], the direct thrombin inhibitor dabigatran etexilate is tested in preclinical fibrosis models [38, 80] and in patients with scleroderma-associated interstitial lung disease [81], and blocking S1P signalling is also considered as an attractive strategy in fibrotic disorders [82]. However, the potential of antagonists of a single GPCR might be limited, as other YAP-activating ligands and receptors can drive the pathogenic processes at the same time. For this reason, direct YAP inhibitors would be more efficacious. Alternatively, our findings in this study and in [18] provide rationale for the use of GPCR agonists of G_αs_-coupled receptors as treatments that actively trigger YAP/TAZ inactivation and thus will have an antifibrotic potential. This is supported by reports that activating IP receptor by selexipag, treprostinil and iloprost [18, 83–89], β2-adrenoreceptors by oladaterol [90], or melatonin receptors by melatonin [91] is antifibrotic *in vitro* and in animal fibrosis models, and at the same time inhibits YAP/TAZ [18, 23, 91].

Our study contributes to a better understanding of fibrosis as a complex interplay of signalling pathways which act in parallel or synergize to induce the pathological processes. We describe how TGFβ1 and GPCR pathways act in concert to drive the profibrotic processes via the regulation of YAP/Smad-dependent transcription. These findings are clinically relevant for understanding the pathogenesis of fibrotic disorders and have implications for development of novel antifibrotic treatments.

## Supporting information

S1 Fig Analysis and validation of the gene expression microarray data. (A, B) NHDF were starved and pretreated with vehicle or 1 μM EW-7197 and then stimulated with 2ng/ml TGFβ1, 1μM LPA, 1μM S1P, 1mU/ml thrombin alone or in combination for 24h. ECM synthesis was measured by ^3^H-proline incorporation (n = 3) and PAI-1 in the cell supernatant was determined by ELISA (n = 3). Mean+/- SEM. (C) NHDF were treated as in A for 3h. CTGF expression was determined by RT-qPCR (n = 3). Mean+/- SEM. Statistics for A-C is summarized in S6 Table. (D) Gene set enrichment analysis (GSEA) of microarray data sets from NHDF reveals enrichment of YAP target genes in NHDF stimulated with 5ng/ml TGFβ1+1μM LPA compared to vehicle-treated cells, 5ng/ml TGFβ1 compared to vehicle-treated cells and 1μM LPA-treated cells compared to vehicle control. ES, enrichment score. (E) Human dermal fibroblasts were starved and stimulated with 5ng/ml TGFβ1, 1μM LPA alone or in combination for 3h. Gene expression was determined by RT-qPCR (n = 3). Mean+/- SEM.
(PDF)

S2 Fig Heatmap of the 972 genes significantly modulated by TGFβ1 versus vehicle for the three conditions LPA, TGFβ1 and LPA+TGFβ1. Genes were sorted by the modulating effect of LPA on the TGF**β**1 effect. For 147 genes (15.1%), addition of LPA further enhanced the effect of TGF**β**1 (>1.5fold increased modulation in combi vs TGF**β**1 alone). For 154 genes (15.8%) LPA addition antagonized the effect of TGF**β**1 (>1.5fold decreased modulation in combi versus TGF**β**1 alone) and for 671 genes (69%) LPA addition on top of TGF**β**1 did not give strong additional modulation (<1.5fold increase or decrease in combi vs TGF**β**1 alone). Details on categorization are shown in S4 Table.
(PDF)

S3 Fig GPCR ligands activate YAP to enhance TGFβ1 response. **(A)** NHDF were treated with 1μM LPA for indicated time. The nuclear intensity of YAP was analyzed by high content imaging of cells stained with anti-YAP antibody. Results were normalized to vehicle-treated cells. Example images are shown on the right (n = 3). Mean+/- SEM. **(B)** NHDF were treated with 5 ng/ml TGFβ1 for 1h. The nuclear intensity of YAP was analyzed by high content imaging of cells stained with anti-YAP antibody. Results were normalized to vehicle-treated cells. Example images are shown on the right (n = 12). (C) NHDF were starved, pretreated with vehicle or 1 μM EW-7197 and then stimulated with 2ng/ml TGFβ1, 1μM LPA, 1μM S1P, 1mU/ml thrombin alone or in combination for 30 min. Whole cell lysates were subjected to immunoblotting. The signal for pSmad3 and pYAP was measured by image densitometry, normalized to the Smad3 or YAP signal and expressed as relative value compared to vehicle-treated sample. (D) Smad2 was immunoprecipitated from nuclear fractions of NHDF stimulated with TGFβ1 alone or TGFβ1 with 1μM LPA for 1h. Samples were analyzed by western blot for the presence of YAP and Smad2. Input represents 10% of the nuclear lysate (TGFβ1 sample) used for immunoprecipitation. The signal for YAP was measured by image densitometry, normalized to Smad2 signal and expressed as relative value compared to TGFβ1-treated sample (n = 2). (E) Human dermal fibroblasts were starved and stimulated for 3h with an increasing dose of TGFβ1 alone or in combination with 1μM LPA, 1μM S1P or 1mU/ml thrombin. PMEPA1, CLDN4 and EGR2 expression was determined by RT-qPCR (n = 3). Mean+/- SEM.
(PDF)

S4 Fig Correlation of YAP nuclear levels with the response to TGFβ1. (A) NHDF were starved and then treated with 5ng/ml TGFβ1 alone or in combination with indicated molecules; 2ng/ml Rho inhibitor (inhib), 500nM latrunculin B (lat B), 10μM forskolin and 50mM 2-DG. αSMA levels were determined by western blot. The signal for αSMA was measured by image densitometry and expressed as relative value compared to vehicle-treated sample (n = 3). Mean+/- SEM. (B) NHDF were starved and then treated with 5ng/ml TGFβ1 alone or in combination with indicated molecules; concentration-response curve (CRC) of LPA and S1P (10-fold dilutions starting at 1μM), thrombin (10-fold dilutions starting at 1mU/ml), 500nM latrunculin B (lat B), 2ng/ml Rho inhibitor (inh), 10μM forskolin, 50mM 2-DG or 1ng/ml Rho activator (act). Integrated nuclear intensity of YAP was analysed after 1h by high content imaging analysis of cells stained against YAP and expressed as fold change (FC) versus TGFβ1-only treated cells. The same treatments were done for 3h to measure gene expression by RT-qPCR. Gene expression was correlated (linear correlation) with YAP nuclear staining (n = 3–4; YAP imaging). (n = 3; RT-qPCR). Mean+/- SEM.
(PDF)

S1 Table Contains expression data of 972 probes which were significantly modulated by TGFβ1 versus vehicle for the three conditions LPA, TGFβ1 and LPA+TGFβ1. For 147 genes (15.1%), addition of LPA further enhanced the effect of TGF**β**1 (>1.5fold increased modulation in combi vs TGF**β**1 alone). For 154 genes (15.8%) LPA addition antagonized the effect of TGF**β**1 (>1.5fold decreased modulation in combi versus TGF**β**1 alone) and for 671 genes (69%) LPA addition on top of TGF**β**1 did not give strong additional modulation (<1.5fold increase or decrease in combi vs TGF**β**1 alone).
(XLSX)

S2 Table Contains expression data for Group1 probes, their annotation as YAP/TAZ target genes and synergistic/additive/antagonistic effects of the LPA/ TGFβ1 combination treatment. (XLSX)

S3 Table Contains expression data for Group 2 probes. (XLSX)

S4 Table Contains expression data for Group 3 probes. (XLSX)

S5 Table Calculated EC_50_ and E_max_ values of TGFβ1 for the induction of PAI-1, CTGF or EDN1 (Fig 2E–2G). (DOCX)

S6 Table Contains statistics of Fig 1A–1C, S1A–S1C Fig and Fig 2D. (DOCX)
