# Supplementary figures and images for: GPCR-induced YAP activation sensitizes fibroblasts to profibrotic activity of TGFβ1
Source: PLoS One. 2020 Feb 13;15(2):e0228195. doi: 10.1371/journal.pone.0228195 (PMC7018035; doi:10.1371/journal.pone.0228195)

**A**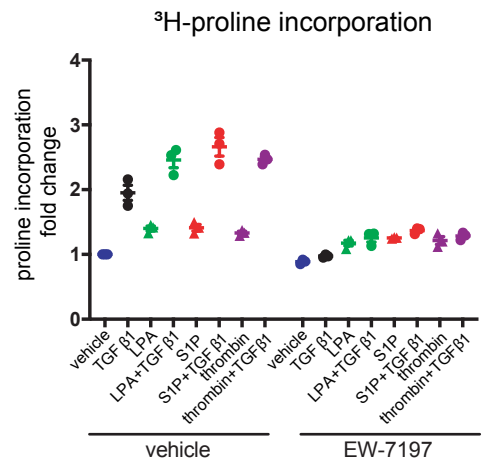**B**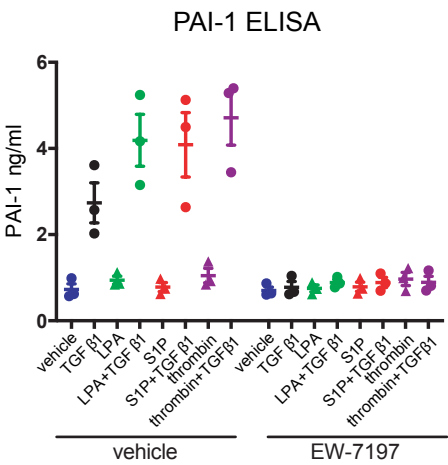**C**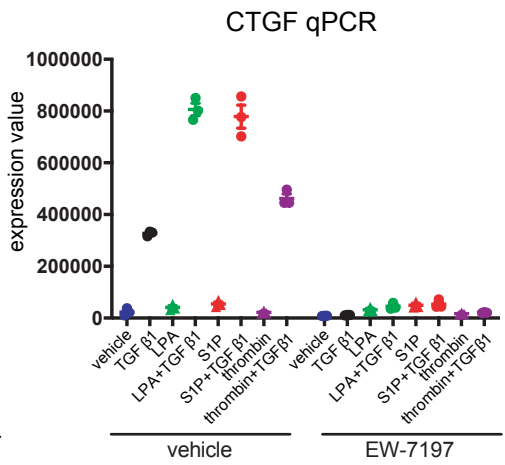**D**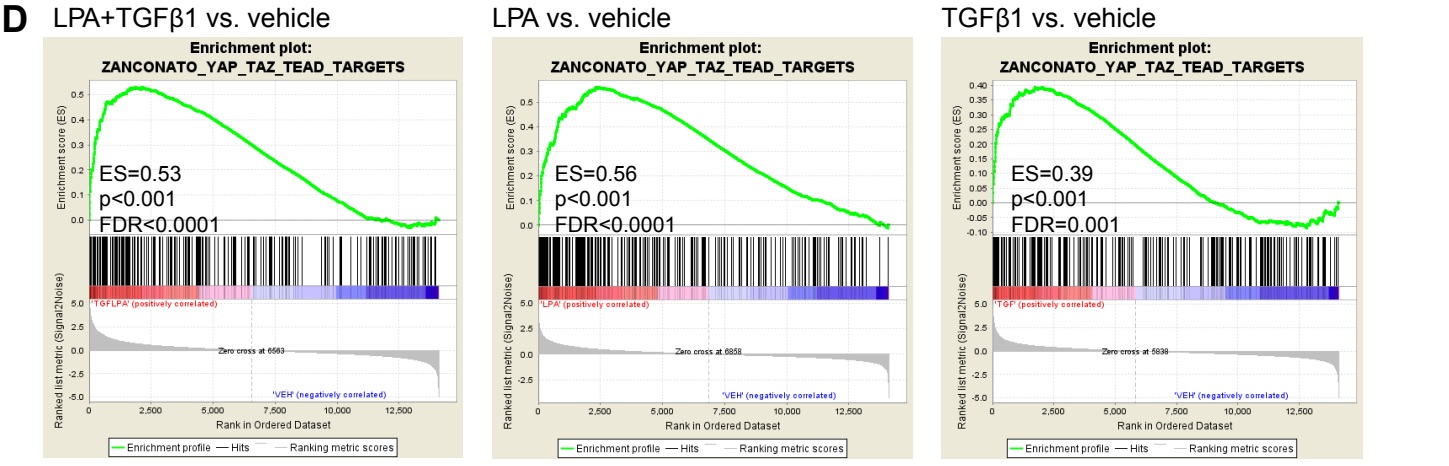**E**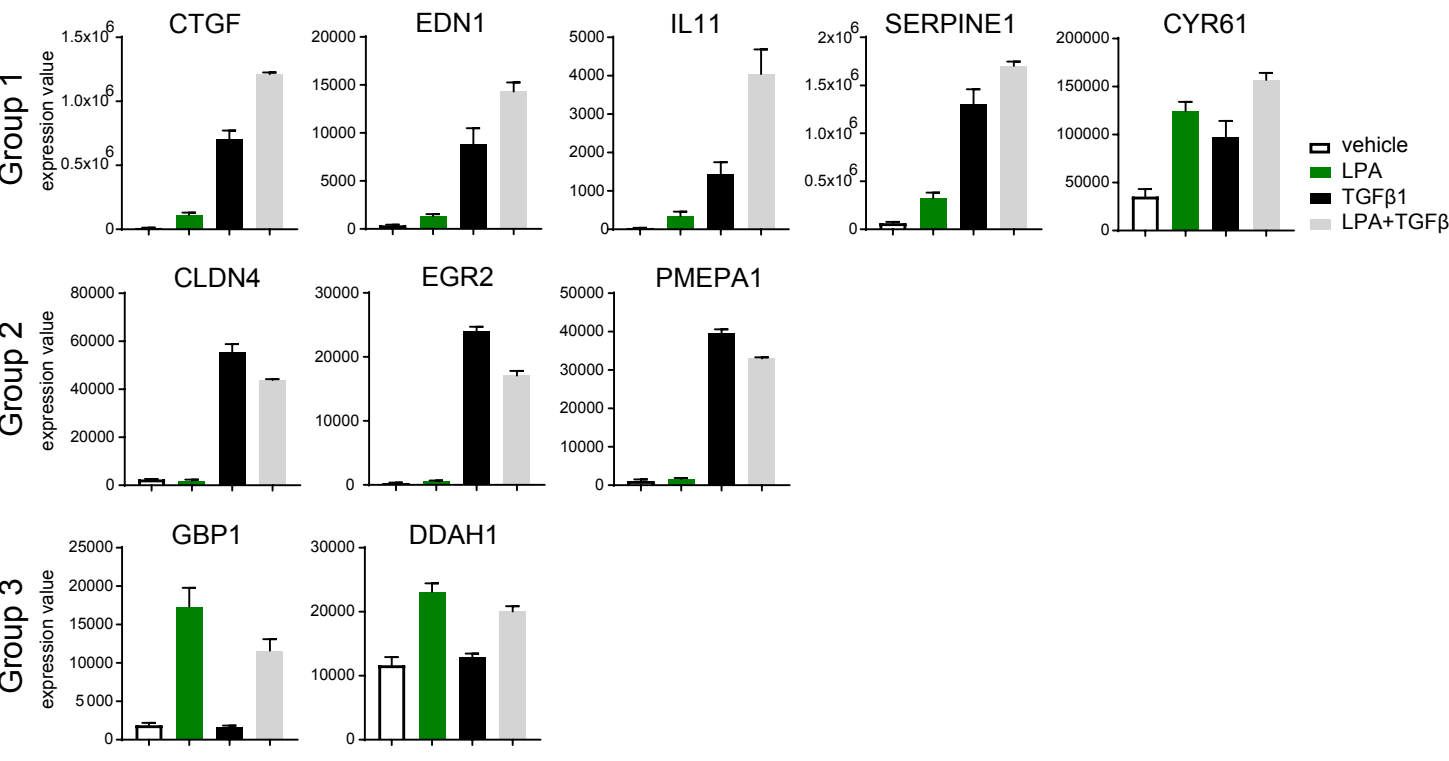

Supplement: S1 Fig — (A, B) NHDF were starved and pretreated with vehicle or 1 μM EW-7197 and then stimulated with 2ng/ml TGFβ1, 1μM LPA, 1μM S1P, 1mU/ml thrombin alone or in combination for 24h. ECM synthesis was measured by 3H-proline incorporation (n = 3) and PAI-1 in the cell supernatant was determined by ELISA (n = 3). Mean+/- SEM. (C) NHDF were treated as in A for 3h. CTGF expression was determined by RT-qPCR (n = 3). Mean+/- SEM. Statistics for A-C is summarized in S6 Table. (D) Gene set enrichment analysis (GSEA) of microarray data sets from NHDF reveals enrichment of YAP target genes in NHDF stimulated with 5ng/ml TGFβ1+1μM LPA compared to vehicle-treated cells, 5ng/ml TGFβ1 compared to vehicle-treated cells and 1μM LPA-treated cells compared to vehicle control. ES, enrichment score. (E) Human dermal fibroblasts were starved and stimulated with 5ng/ml TGFβ1, 1μM LPA alone or in combination for 3h. Gene expression was determined by RT-qPCR (n = 3). Mean+/- SEM. (PDF) [file pone.0228195.s001.pdf]

LPA

TGFβ1

TGFβ1+LPA

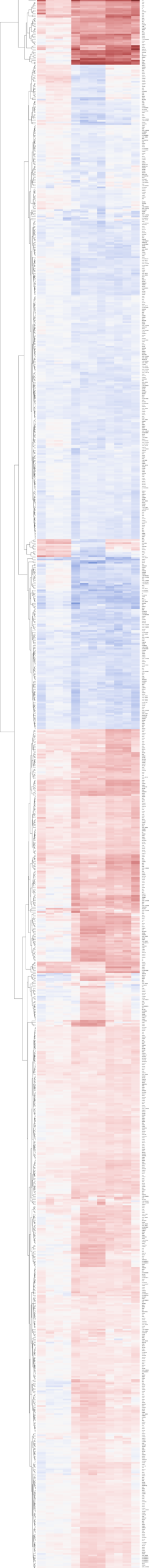

Supplement: S2 Fig — Genes were sorted by the modulating effect of LPA on the TGFβ1 effect. For 147 genes (15.1%), addition of LPA further enhanced the effect of TGFβ1 (>1.5fold increased modulation in combi vs TGFβ1 alone). For 154 genes (15.8%) LPA addition antagonized the effect of TGFβ1 (>1.5fold decreased modulation in combi versus TGFβ1 alone) and for 671 genes (69%) LPA addition on top of TGFβ1 did not give strong additional modulation (<1.5fold increase or decrease in combi vs TGFβ1 alone). Details on categorization are shown in S4 Table. (PDF) [file pone.0228195.s002.pdf]

**A**

YAP  
immunofluorescence

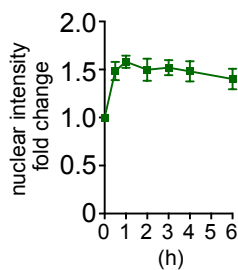**B**

YAP  
immunofluorescence

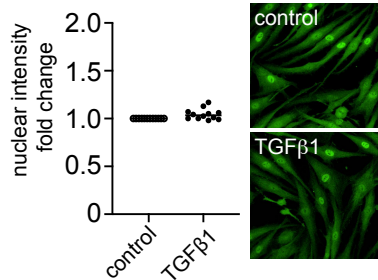**C**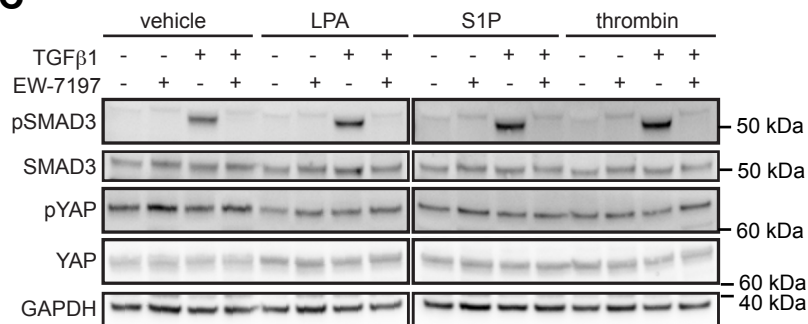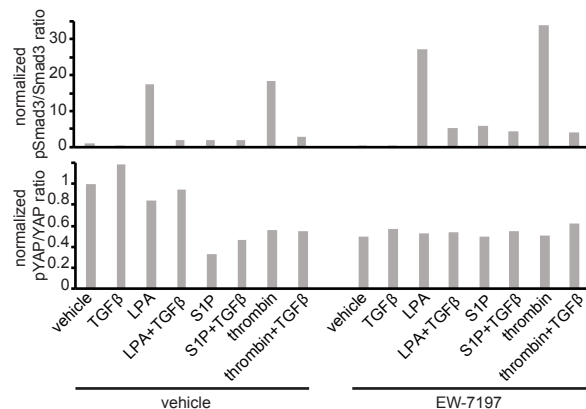**D**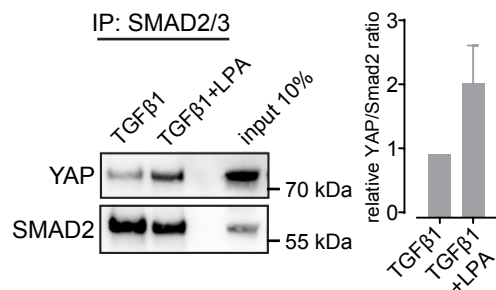**E**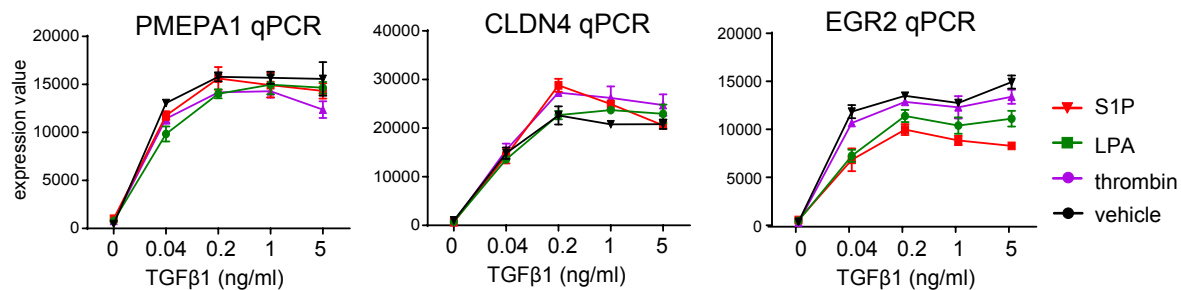

Supplement: S3 Fig — (A) NHDF were treated with 1μM LPA for indicated time. The nuclear intensity of YAP was analyzed by high content imaging of cells stained with anti-YAP antibody. Results were normalized to vehicle-treated cells. Example images are shown on the right (n = 3). Mean+/- SEM. (B) NHDF were treated with 5 ng/ml TGFβ1 for 1h. The nuclear intensity of YAP was analyzed by high content imaging of cells stained with anti-YAP antibody. Results were normalized to vehicle-treated cells. Example images are shown on the right (n = 12). (C) NHDF were starved, pretreated with vehicle or 1 μM EW-7197 and then stimulated with 2ng/ml TGFβ1, 1μM LPA, 1μM S1P, 1mU/ml thrombin alone or in combination for 30 min. Whole cell lysates were subjected to immunoblotting. The signal for pSmad3 and pYAP was measured by image densitometry, normalized to the Smad3 or YAP signal and expressed as relative value compared to vehicle-treated sample. (D) Smad2 was immunoprecipitated from nuclear fractions of NHDF stimulated with TGFβ1 alone or TGFβ1 with 1μM LPA for 1h. Samples were analyzed by western blot for the presence of YAP and Smad2. Input represents 10% of the nuclear lysate (TGFβ1 sample) used for immunoprecipitation. The signal for YAP was measured by image densitometry, normalized to Smad2 signal and expressed as relative value compared to TGFβ1-treated sample (n = 2). (E) Human dermal fibroblasts were starved and stimulated for 3h with an increasing dose of TGFβ1 alone or in combination with 1μM LPA, 1μM S1P or 1mU/ml thrombin. PMEPA1, CLDN4 and EGR2 expression was determined by RT-qPCR (n = 3). Mean+/- SEM. (PDF) [file pone.0228195.s003.pdf]

**A****NHDF**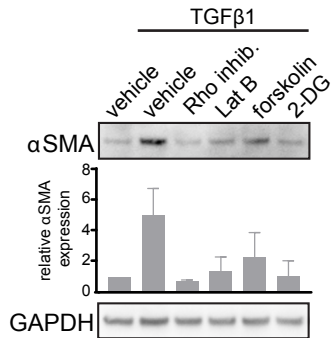**B****NHDF**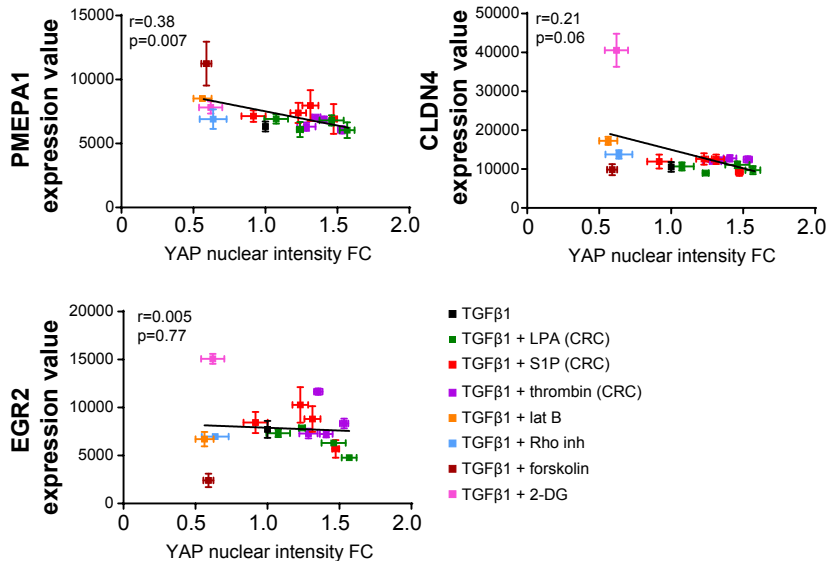

Supplement: S4 Fig — (A) NHDF were starved and then treated with 5ng/ml TGFβ1 alone or in combination with indicated molecules; 2ng/ml Rho inhibitor (inhib), 500nM latrunculin B (lat B), 10μM forskolin and 50mM 2-DG. αSMA levels were determined by western blot. The signal for αSMA was measured by image densitometry and expressed as relative value compared to vehicle-treated sample (n = 3). Mean+/- SEM. (B) NHDF were starved and then treated with 5ng/ml TGFβ1 alone or in combination with indicated molecules; concentration-response curve (CRC) of LPA and S1P (10-fold dilutions starting at 1μM), thrombin (10-fold dilutions starting at 1mU/ml), 500nM latrunculin B (lat B), 2ng/ml Rho inhibitor (inh), 10μM forskolin, 50mM 2-DG or 1ng/ml Rho activator (act). Integrated nuclear intensity of YAP was analysed after 1h by high content imaging analysis of cells stained against YAP and expressed as fold change (FC) versus TGFβ1-only treated cells. The same treatments were done for 3h to measure gene expression by RT-qPCR. Gene expression was correlated (linear correlation) with YAP nuclear staining (n = 3–4; YAP imaging). (n = 3; RT-qPCR). Mean+/- SEM. (PDF) [file pone.0228195.s004.pdf]
