# Supplementary material for: GPCR-induced YAP activation sensitizes fibroblasts to profibrotic activity of TGFβ1
Source: PLoS One. 2020 Feb 13;15(2):e0228195. doi: 10.1371/journal.pone.0228195 (PMC7018035; doi:10.1371/journal.pone.0228195)
Supplement: S5 Table — (DOCX) [file pone.0228195.s009.docx]

**S5 Table**

|  | **PAI-1** |  |  | **CTGF** |  | **EDN1** |
| --- | --- | --- | --- | --- | --- | --- |
|  | EC50 | Emax (%) |  | Emax (%) |  | Emax (%) |
| veh | 92 pg/ml | 100 |  | 100 |  | 100 |
| LPA | 108 pg/ml | 197 |  | 291 |  | 284 |
| S1P | 164 pg/ml | 179 |  | 256 |  | 547 |
| thrombin | 160 pg/ml | 238 |  | 190 |  | 275 |

Calculated EC_50_ and E_max_ values of TGFβ1 for the induction of PAI-1, CTGF or EDN1 (Fig. 2E-G)
