# Supplementary material for: GPCR-induced YAP activation sensitizes fibroblasts to profibrotic activity of TGFβ1
Source: PLoS One. 2020 Feb 13;15(2):e0228195. doi: 10.1371/journal.pone.0228195 (PMC7018035; doi:10.1371/journal.pone.0228195)
Supplement: S6 Table — (DOCX) [file pone.0228195.s010.docx]

**S6 Table**

Contains statistics of Fig 1A-C, S1 Fig A-C and 2D.

|  |  |  |  |  |  |  |  |
| --- | --- | --- | --- | --- | --- | --- | --- |
| Fig 1A | TGFβ1 | LPA | TGFβ1+LPA | S1P | TGFβ1+S1P | thrombin | TGFβ1+ thrombin |
| Sample vs vehicle | * | * |  | * |  | * |  |
| Sample vs TGFβ1 |  |  | * |  | ** |  | *** |
|  |  |  |  |  |  |  |  |
| Fig 1B | TGFβ1 | LPA | TGFβ1+LPA | S1P | TGFβ1+S1P | thrombin | TGFβ1+ thrombin |
| Sample vs vehicle | *** | ns |  | ns |  | * |  |
| Sample vs TGFβ1 |  |  | *** |  | ** |  | *** |
|  |  |  |  |  |  |  |  |
| Fig 1C | TGFβ1 | LPA | TGFβ1+LPA | S1P | TGFβ1+S1P | thrombin | TGFβ1+ thrombin |
| Sample vs vehicle | ** | ns |  | *** |  | *** |  |
| Sample vs TGFβ1 |  |  | *** |  | *** |  | *** |
|  |  |  |  |  |  |  |  |
| S1A Fig | TGFβ1 | LPA | TGFβ1+LPA | S1P | TGFβ1+S1P | thrombin | TGFβ1+ thrombin |
| Sample vs vehicle | * | ** |  | * |  | ** |  |
| Sample vs TGFβ1 |  |  | * |  | * |  | * |
|  |  |  |  |  |  |  |  |
| S1B Fig | TGFβ1 | LPA | TGFβ1+LPA | S1P | TGFβ1+S1P | thrombin | TGFβ1+ thrombin |
| Sample vs vehicle | * | ns |  | ns |  | ns |  |
| Sample vs TGFβ1 |  |  | ns |  | ns |  | ns |
|  |  |  |  |  |  |  |  |
| S1C Fig | TGFβ1 | LPA | TGFβ1+LPA | S1P | TGFβ1+S1P | thrombin | TGFβ1+ thrombin |
| Sample vs vehicle | *** | ns |  | ns |  | ns |  |
| Sample vs TGFβ1 |  |  | *** |  | *** |  | ** |
|  |  |  |  |  |  |  |  |
| Fig 2D | TGFβ1 | LPA | TGFβ1+LPA | S1P | TGFβ1+S1P | thrombin | TGFβ1+ thrombin |
| Sample vs vehicle | ** | ** |  | * |  | ns |  |
| Sample vs TGFβ1 |  |  | * |  | * |  | * |
